# Estimating the growth parameters, exploitation rate, biomass and maximum sustainable yield of long whisker catfish *Mystus gulio* (Hamilton, 1822) in the coastal waters from southwestern Bangladesh

**DOI:** 10.1016/j.heliyon.2024.e29788

**Published:** 2024-04-16

**Authors:** Obaidur Rahman, Taiba Akter Laboni, Mst. Shahinur Khatun, Md. Ashekur Rahman, Md. Akhtarul Islam, Md. Mizanur Rahman, Most. Farida Parvin, Md. Joynal Abedin, Md. Yeamin Hossain

**Affiliations:** a Department of Fisheries, University of Rajshahi, Rajshahi, 6205, Bangladesh; b Institute of Natural Resources Research and Development, Rajshahi, 6206, Bangladesh; c Institute of Environment Sciences, University of Rajshahi, Rajshahi, 6205, Bangladesh; d Department of Zoology, Carmichael College, National University, Bangladesh

**Keywords:** Biomass, Coastal waters, Exploitation, Growth parameters, *Mystus gulio*, Mortality, Maximum sustainable yield

## Abstract

The research provides a comprehensive analysis of *Mystus gulio* including growth pattern, growth parameters, recruitment patterns, mortality rates, biomass, exploitation rate (*E*), and the estimation of maximum sustainable yield (*MSY*) within the southwestern coastal waters of Bangladesh. From January to December 2017, fishers provided around 1200 specimens. FAO-ICLARM Stock Assessment Tool and Excel-add-in-solver were used to assess stock status through length-frequency data. Indeed, the research findings indicated that the population of *M. gulio* displayed negative allometric for both individuals (*b* = 2.53 for male, *b* = 2.50 for female), as demonstrated by the calculated allometric coefficient value. Nonetheless, the population's dynamic characteristics revealed an asymptotic length (*L*_*∞*_) of 19.34 cm, 23.28 cm and growth coefficient (*K*) 0.94 year^−1^ and 0.81 year^−1^ for male and female *M. gulio*. The growth performance indexes (Ø') of 2.55 and 2.64 for male and female and maximum lifespan (*t*_*max*_) 3.20 years and 3.70 years respectively. This study revealed that the slightly variations in the natural mortality rate (*M*) for both specimens at 1.55 year^−1^ and 1.59 year^−1^. The fishing mortality rate (*F*) 2.75 year^−1^ and 1.98 year^−1^and total mortality rate (*Z*) 4.30 year^−1^ and 3.57 year^−1^ for male and females, respectively. The maximum permissible exploitation rate (*E*_*max*_ = 0.421) was lower than the actual exploitation rate (*E* = 0.63). The *MSY* was calculated at 67.968 metric tons. Without a doubt, overfishing stands out as the most critical threat to the wild stock. Therefore, it is clear that the existing fishing approach was not efficiently managed the standing stock in a sustainable manner. The findings would be useful for established proper fishing regulations in coastal waters and the surrounding ecosystems.

## 1 Introduction

The mounting demand from consumers, especially in open-water ecosystems, has subjected a significant portion of natural stock to immense fishing pressure [1]. Fish, acknowledged as a renewable resource, currently face restrictions due to this situation [2]. There are over 4100 distinct species of catfish, which is the third biggest teleost fish species after Cypriniformes and Perciformes and makes up about 12 % of all bony fishes [3,4]. These species are recognized as an exceptional source of crucial macro- and micronutrients. They are essential for reducing the malnutrition issue in Bangladesh [5]. The rural inhabitants across various South Asian nations, including Bangladesh, catfishes such as *Mystus cavasius*, *Rita rita*, *Clupisoma garua*, *Eutropiichthys vacha*, and *Ompok pabda* is a staple in the diets owing to its exceptional nutritional value, encompassing rich protein content, essential micro and macronutrients, as well as a diverse array of vitamins and minerals [6]. These specific species are highly sought after by both small and large-scale fishers in Bangladesh, given their elevated market value and strong consumer preferences. Traditional fishing gears are employed by these fishers in their pursuit [7,8]. The nutrient composition of catfish highlights its high nutritional content, characterized by abundant protein, low fat and cholesterol levels, and a rich source of certain vitamins and minerals. Notably, catfish is particularly high in Vitamin-D content. Furthermore, catfish is a significant source of omega-3 fatty acids, which are known to protect against a variety of severe non-communicable illnesses, including cardiovascular disease. Catfish's composition is made up of 18.43 % protein, 68.80 % fat, and 22.40 % moisture. Notably, catfish contains essential omega-3 fatty acids. Interestingly, research reveals that inland fish, like catfish, exhibit higher omega-3 fatty acid content compared to marine fish like sardine [9].

The Long Whisker catfish, scientifically known as *Mystus gulio* (Siluriformes: Bagridae), is a versatile fish species inhabiting freshwater, brackish, and anadromous environments [10]. Although commonly referred to as the "river catfish," this species assumes various names across different nations, including Nona tengra in Bangladesh, Nga-zin in Myanmar, and long whisker catfish in India and Sri Lanka. In India, this particular species is known by various names, including kala tenguah, kontia and shingat [11]. *M. gulio* stands out as a commercially significant food fish in numerous Asian nations. Its exceptional taste has led to widespread consumer favor, resulting in a substantial market demand, particularly in the regions of Eastern India and Bangladesh. The distribution of *M. gulio* throughout Bangladesh's coastal waters as well as those of the nations that border the eastern Indian Ocean, from India to Indonesia and Vietnam. There have been reports of its presence in Pakistan as well [12]. This catfish is unique in that it is a brackish water species that ventures into and it also ability to thrive in brackish water habitats, as well as entering tidal rivers in Bangladesh and the Bay of Bengal [13]. It is commonly found in estuaries, tidal rivers, lakes, *beels*, *haors* and rivers [14]. *M. gulio* primarily preys on juvenile organisms, while adult individuals of this species consume a varied diet that includes debris, zooplankton, zoobenthos, other benthic invertebrates, fish eggs, and larvae [15], this species is regarded as being of least concern in Bangladeshi water bodies [16].

To ensure the sustainable management and protection of wild fish stocks, a variety of life-history parameters, including growth, reproductive characteristics, recruitment patterns, and mortality rates, must be evaluated [17]. Examining the growth pattern of fish is a common practice aimed at observing fluctuations in growth and condition indices across different seasons. Moreover, the analysis of growth patterns in fish populations plays a key role in accurately calculating both the production and biomass of these populations. Dynamic mathematical models [18] are frequently used to create efficient management methods for predicting forthcoming yields and stock biomass while considering various fishing strategies; these models are extensively employed to formulate effective management approaches [19].

These models help to developed with a complete understanding of variables including growth, recruitment, mortality, exploitation rates, relative yield per recruit, and maximum sustainable yield, all of which are essential for understanding and effectively managing fish populations. The challenges in precisely determining fish age might be partly responsible for the limited use of dynamic pool models in influencing fisheries management plans in tropical and sub-tropical environments. However, introduce of length-based stock assessment methodologies has opened up the possibility to explore the details of fish stock population dynamics in tropical waters, overcoming previous limitations in understanding these ecosystems [20,21].

Research on the stock assessment of *M. gulio* in the present location has been notably scarce. Nevertheless, available evidence is limited but does touch upon various dimensions of *M. gulio*, encompassing elements such as length weight relationships; age, growth and survival; food, feeding habit and stocking density; disease; heavy metal (Table 1). Therefore, the objective of this research was to comprehensively analyze several aspects of stock assessment for *M. gulio*, encompassing growth patterns, growth parameters, recruitment dynamics, mortality rates, exploitation levels, maximum sustainable yield, and relative yield per-recruit using consecutive 12-month samples, covering a range of sizes from small to large every month.

**Table 1:** Available studies on the Long Whisker cat fish *Mystus gulio* in different waterbodies (south Asian countries).

Aspects	Water body/country	References
Length-weight relationship	West Bengal, India	Dasgupta [22]
	Indian Sub- Continent	Begum et al. [23]
	Ganges River (NW Bangladesh)	Hossain et al. [24]
	Chilika Lagoon, India	Panda et al. [25]
	Coastal water, Satkhira, Bangladesh	Rahman et al. [26]
	Coastal water, Satkhira, Bangladesh	Rahman et al. [27]
	Brakish water, Bangladesh	Pujiah et al. [28]
Age, growth and survival	Hooghly estuary, India	Pantulu [29]
	Estuarine catfish, Bangladesh	Islam et al. [30]
Food, Feeding habit and stocking density	Estuarine cat fish, India	David [31]
	Brackish water, Bangladesh	Sarker et al. [32]
	Bangladesh	Begum et al. [33]
	Bangladesh	Begum et al. [34]
	Bangladesh	Begum et al. [35]
	Nursery ponds, Bangladesh	Paikgacha [36]
	Brackish water catfish, Bangladesh	Siddiky et al. [37]
	East Godavari district (Andhra Pradesh)	Rao [38]
	Bangladesh coast	Hossain et al. [39]
	Fry reared in net cage system, Bangladesh	Biswas et al. [40]
	Lake Pulicat, India	Kaliyamurthy [41]
	Estuarine catfish, India	Lal et al. [42]
	Brakish water, Bangladesh	Kumar et al. [43]
	Brakish water, Bangladesh	Kumar et al. [44]
	Estuarine catfish, Bangladesh	Alam et al. [45].
Disease	West Bengal, India	Guchhait et al. [46]
Heavy metal	Bolgoda Lake, Sri Lanka	Senarathne and Pathiratne [47]

## 2 Materials and methods

### 2.1 Sampling area

**Fig. 1:**
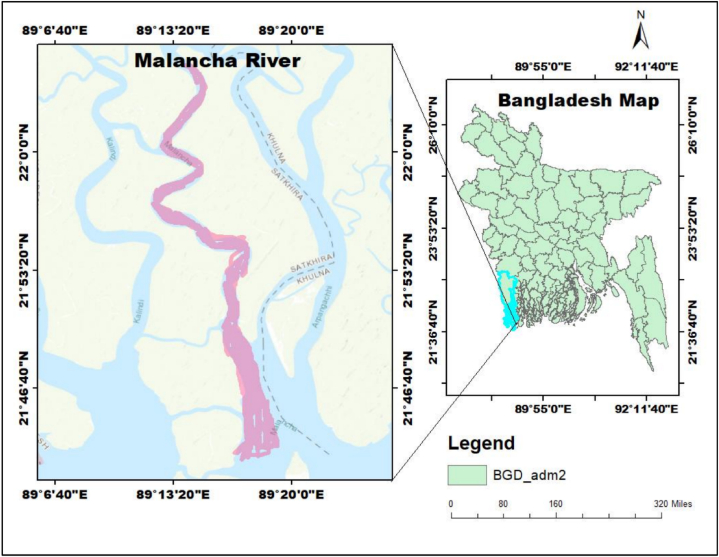
The study site in coastal waters (Malancha River) in Satkhira, southwestern, Bangladesh.

A number of 1200 individuals of *M. gulio* were gathered from the fishers that was caught at various sites of coastal waters (Malancha River) in Satkhira, southwestern Bangladesh (Latitude 21°58'31.26"N, Longitude 89°14'48.46"E) (Fig. 1). Throughout the sampling period, samples of *M. gulio* were captured in the month of January to December 2017, employing conventional fishing techniques and gear, including gill nets and trawls, mesh size ranging from 1 to 2 cm. Once collected, the specimens were immediately preserved in a solution of 10 % buffered formalin and thereafter stored on ice, ensuring their optimal condition for any potential subsequent investigations.

### 2.2 Fish measurement and length frequency distribution (LFD)

Afterwards arrival in the lab, each specimen's TL (Total Length) in cm and BW (Body Weight) in g were measured utilizing electronic balances and digital slide, calipers with an accuracy of 0.01 cm and 0.01 g, individually. For this study, males and females were identified based on morphometric characteristics. The monthly length frequency distribution (LFD) for the *M. gulio* species was analyses using excel and R statistical software.

### 2.3 Growth pattern

Using the formula $BW{=aL}^{b}$ BW, represents the body weight (g) and *L* represents the total length (cm), LWRs assessed indicate the growth pattern, the *t*-test was applied. Natural logarithms were utilized to estimate the parameters *a* and *b* in linear regression analyses: ln(W)=ln(a)+bln(L). Additionally, the confidence limits at 95 % and the *r*^*2*^ coefficient of determination were calculated for the variables *a* and *b* respectively for both male and female *M. gulio*. Extreme outliers were expelled by the regression analysis through using the Froese method [48].

### 2.4 Estimation of growth parameters

To ensure precise direct fitting of length-frequency data, to generate a dataset including a time series of information that exhibits a well-defined class size. The Powell-Wetherall plot is utilized for independent estimation of the values of *L* and *Z*/*K* [49]. The values of variable *L* and *Z/K* were calculated by integrating a series of length-frequency data acquired at predefined interims. The input parameter serves as a graphical determination of the minimum length is fully engaged by the gear. (*L'*, or cut-off length) can be represented by a function of the form: $\left(L-L^{\prime }\right)=a+b*L^{\prime }$, here *L* represents the mean length of all fish equal to or longer than the length *L'*, which serves to establish a series of lower limits for the length intervals of fully vulnerable fish, thereby ensuring sustainable practices and promoting conservation efforts. The regression line on the Wetherall plot is determined by fitting it through all the data points representing the fully exploited segment of the sample. This is often achieved by selecting data from LFD that corresponds to one length-interval beyond the highest mode.

The values of *K* and *L*_*∞*_ were estimated for each sex by applying the von Bertalanffy growth equation to the input data, which was segregated according on sex: $L_{t}=L_{\infty }\left[1-\exp\left\{-K\left(t-t_{0}\right)\right\}\right]$ [50], in this context, *L*_*t*_ represents the total length (cm) at a certain age *t* (months). *L*_*∞*_ denotes the asymptotic total length (cm), *K* represents the growth coefficient (years^−1^) and *t*_*0*_ signifies the hypothetical age at which the total length would be zero. The estimated value of *Z/K* was calculated from the regression line of slope, *b* as: z/k=−(1+b)/b. Additionally, *K* was determined through model [20]: $K=3/T_{\max}$ and *t*_*0*_ was calculated as $\log\left({-t}_{0}\right)=-0.3922-0.2752\;\log\;L_{\infty }-1.038\;\log\;K$ [51]. The method was used to determine the age at first sexual maturity (*t*_*m*_), $t_{m}\left(50\%\right)=\left(-1/1\right)\ln\left(1-L_{m}/L_{\infty }\right)$. Growth performance index was estimated as ${\emptyset^{\prime }=\log}_{10}\;K+2\;\log_{10}L_{\infty }$ [20].

### 2.5 Size at first sexual maturity (*L*_*m*_)

This equation log (*L*_*m*_) = −0.1189 + 0.9157* log (*L*_*max*_) [52] was used to figure out the *L*_*m*_ of *M. gulio* in the coastal water, southwestern, Bangladesh.

### 2.6 Longevity

The probable lifespan of *M. gulio* was estimated through the application of a specialized formula [20] $T_{\max}=3/K$ , *L* and *K* represent the von Bertalanffy growth parameter. Taylor [53] defined longevity (*A*) as the time required to attain 95 % of the *L*_∞_ with the following equation: $A_{95\%}=t_{0}+\frac{\log_{e}\left(1-0.95\right)}{k}$. This equation was used to determine longevity based on 99 % of *L*_∞_ by substituting 0.99 for 0.95 in the equation [53].

### 2.7 Estimation of mortality and exploitation

The length-converted catch curve equation was using to determine the instantaneous rate of total mortality *(Z)* for *M. gulio* in coastal waters [54]. After implementing, the following proposal was put forward to derive an empirical formula for computing of natural mortality *(M)* for *M. gulio.* $\log_{10}\;M=-0.0152-0.279\;\log_{10}L_{\infty }+0.6543\;\log_{10}\;K+0.4634\;\log_{10}\;T$ [52], where, *M* represent the natural mortality, *L*_*∞*_ denotes the asymptotic total length, *K* is the growth coefficient associated with the von Bertalanffy technique, and *T* represents the ordinary annual ambient temperature, measured in degrees Celsius, within which the stocks under consideration reside. The natural mortality (*M*) of *M gulio* was subtracted from the total mortality (*Z*), which resulted in the calculation of the fishing mortality *(F)* of *M gulio*: *Z = M + F.* The exploitation rate (*E*) of *M. gulio* was determined by calculating the percentage of the fishing mortality compared to the total mortality [55]. The formula for this calculation is as follows: E=F/Z=F/(F+M).

### 2.8 Recruitment pattern

The von Bertalanffy formula was applied to analyses the whole time series of LFDs and growth characteristics in order to identify the recruitment pattern of *M. gulio*. Input parameters *L*_*∞*_*, K*, and *t*_*0*_ were utilized in the analysis [56]. The proportion of recruitment against time in months was used to describe the recruiting pattern. Excel-add-in-solver was used to calculate the recruiting pattern's normal distribution [57].

### 2.9 Relative yield-per-recruit *(Y'/R)* and maximum sustainable yield *(MSY)*

The prediction of the relative yield per recruit *(Y'/R)* for the *M. gulio* species in fisheries was accomplished by employing a model [18] and then apprised [58]. The relative biomass per recruit (*B'/R*) of *M. gulio* was calculated by *E*_*max*_ (The exploitation rate that results in the maximum yield), *E*_*0.1*_ (The exploitation rate at which its virgin stock's 10 % of its marginal *Y'/R* rise occurs), and *E*_*0.5*_ (The exploitation rate at which the stock is diminished to half of its initial virgin biomass signifies a tipping point in resource depletion and underscores the need for careful conservation measures) were calculated utilizing the first component of the formula [18]. The determination of the steady state biomass (SSB) was carried out through the application of the length-structured virtual population analysis (VPA) predictable combined into FiSAT II. The *MSY* was estimated as MSY=0.5SSB*Z.

### 2.10 Temperature measurement

In order to assess the status of water quality of the coastal water, temperature (^o^C), was recorded monthly basis following [59,60] an estimated 27 °C temperature on average was recorded in the Malancha River.

### 2.11 Statistical analyses

Software like FiSAT and the Microsoft Excel add-in Solver was used to conduct statistical analysis. At 5 % (*p* < 0.05), all statistical tests were determined to have been statistically significant.

## 3 Results

### 3.1 Length frequency distribution (LFD)

**Fig. 2:**
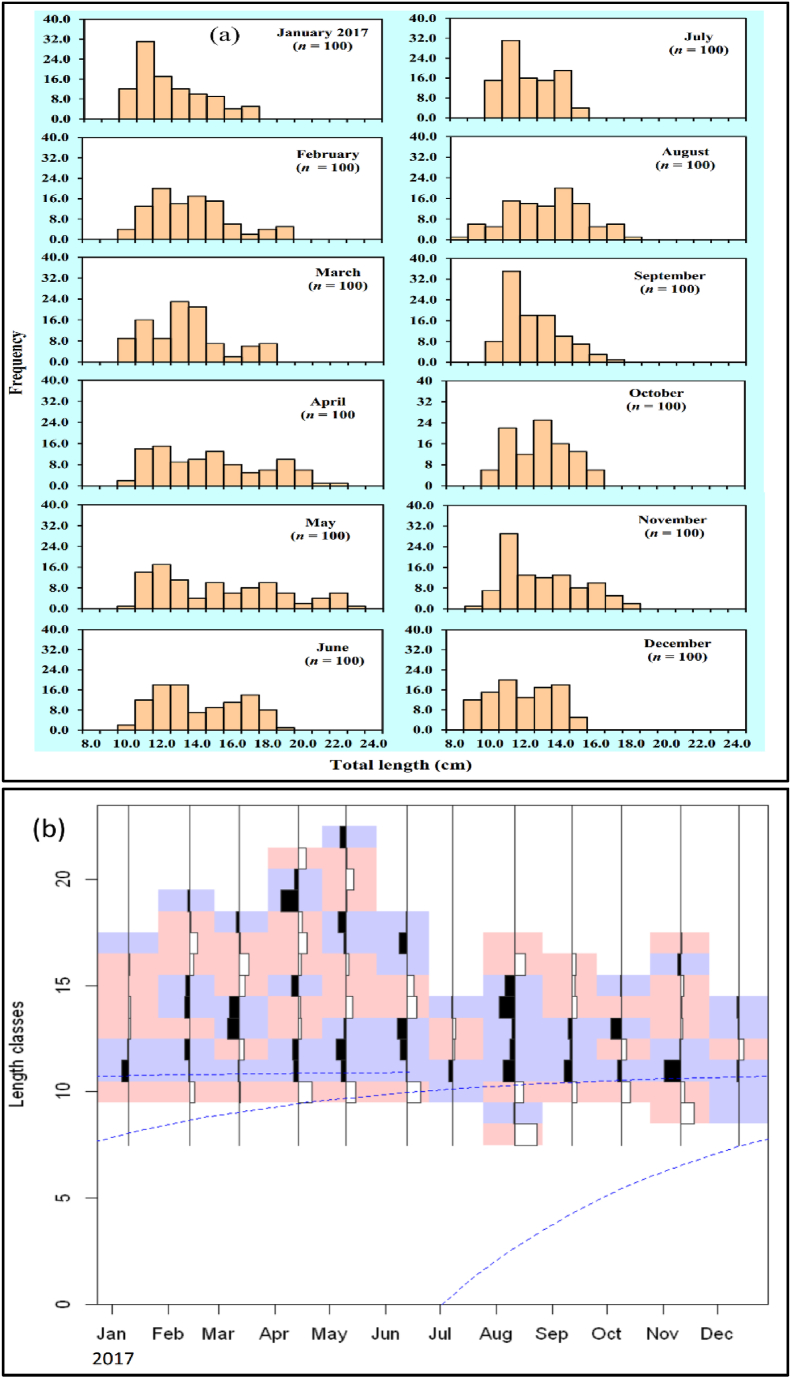
The length-frequency distribution by using (a) Excell and (b) R statistical software of *Mystus gulio* in the coastal waters, southwestern Bangladesh.

LFD exposed that the TL and BW diverse from 7.80 to 22.10 cm and 10.52–128.82g respectively (Fig. 2 (a, b)).

### 3.2 Growth pattern

**Fig. 3:**
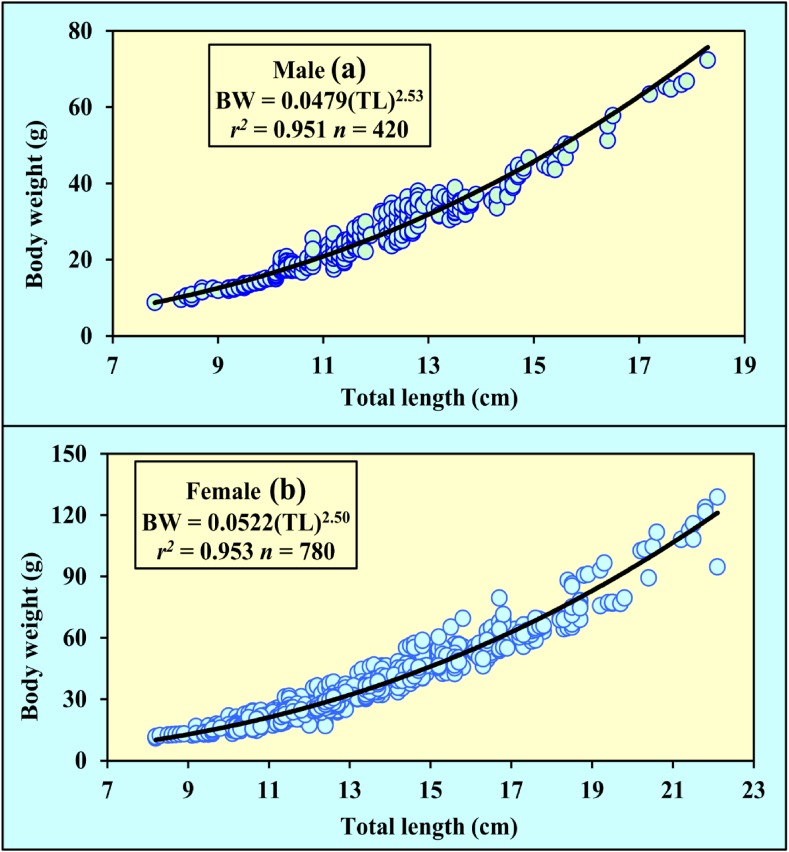
Growth pattern for the (a) male and (b) female of *Mystus gulio* in the coastal waters, southwestern Bangladesh.

Table 2 summarizes the descriptive data for monthly length-weight measurements of *M. gulio* in coastal waters*. M. gulio* LWRs parameters *a* and *b* were determined using length and weight data, and the LWR was computed as BW = 0.0479 TL^2.53^ for male (*p* < 0.0001; *r*^*2*^ = 0.951) and BW = 0.0522 TL^2.50^ for female (*p* < 0.0001; *r*^*2*^ = 0.953) (Fig. 3(a and b)).

**Table 2:** Descriptive statistics on the total length (cm) and body weight (g) measurements of *Mystus gulio* (Hamilton, 1822) in the coastal waters southwestern, Bangladesh.

Month	*n*	Total length (cm)				Body weight (g)			
		Min	Max	Mean ± SD	95 % CL	Min	Max	Mean ± SD	95 % CL
Jan	100	9.30	16.80	11.91 ± 1.99	11.51 to 12.30	14.12	68.98	29.51 ± 13.21	26.89 to 32.13
Feb	100	9.50	18.70	13.13 ± 2.28	12.67 to 13.58	13.1	68.98	32.07 ± 13.95	29.30 to 34.84
Mar	100	9.10	17.50	12.78 ± 2.26	12.33 to 13.22	12.56	67.01	32.42 ± 14.37	29.57 to 35.27
Apr	100	9.50	21.20	14.32 ± 3.09	13.70 to 14.93	12.50	94.78	41.55 ± 21.67	37.25 to 45.85
May	100	9.90	22.10	14.70 ± 3.54	13.99 to 15.40	11.38	128.82	45.61 ± 32.50	39.16 to 52.06
Jun	100	9.60	18.20	13.59 ± 2.41	13.12 to 14.07	13.50	69.71	37.24 ± 15.97	34.07 to 40.40
Jul	100	9.20	14.80	11.49 ± 1.50	11.19 to 11.79	12.80	41.23	22.98 ± 7.71	21.45 to 24.51
Aug	100	7.80	17.60	12.55 ± 2.25	12.10 to 13.00	10.52	69.56	34.33 ± 14.90	31.38 to 37.29
Sep	100	9.50	16.70	11.72 ± 1.63	11.39 to 12.04	13.52	79.57	30.12 ± 13.74	27.39 to 32.85
Oct	100	9.00	15.60	12.30 ± 1.72	11.95 to 12.64	12.64	52.50	28.29 ± 10.72	26.17 to 30.42
Nov	100	8.90	17.20	12.37 ± 2.19	11.93 to 12.80	12.45	62.54	29.67 ± 13.47	26.99 to 32.34
Dec	100	8.20	14.50	11.29 ± 1.81	10.93 to 11.65	11.09	46.00	24.90 ± 10.24	22.87 to 26.94

*n*, sample size; min, minimum; max, maximum; SD, standard deviation; CL, confidence limit.

### 3.3 Growth parameters

**Fig. 4:**
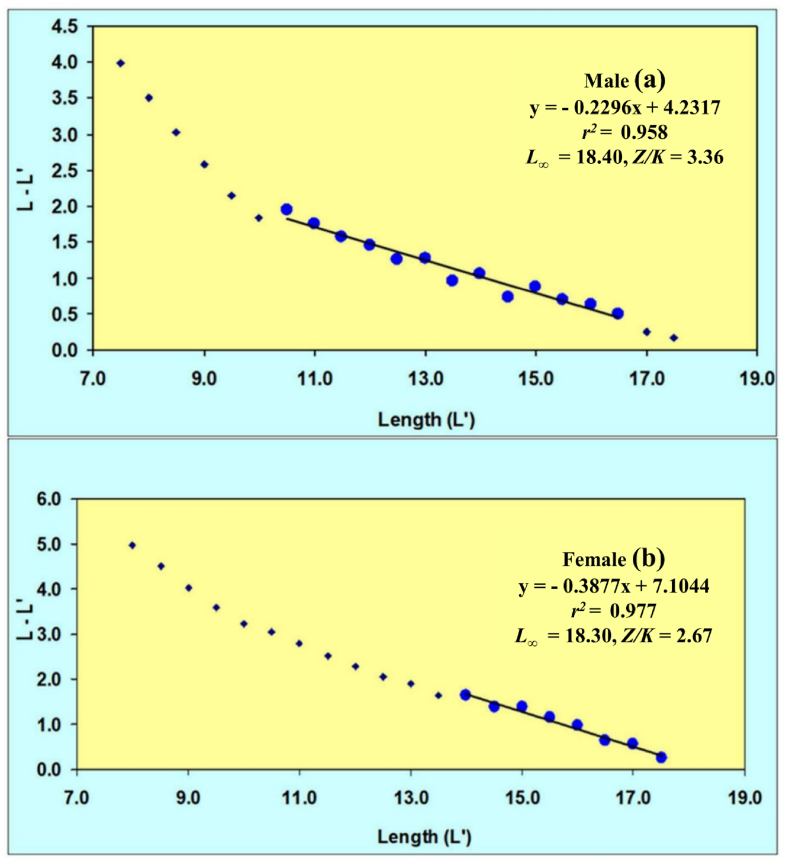
A Powel-Wetherall plot for the (a) male and (b) female of *Mystus gulio*. Solid blue symbols are used in the regression which provides asymptotic TL and *Z/K*.

**Fig. 5:**
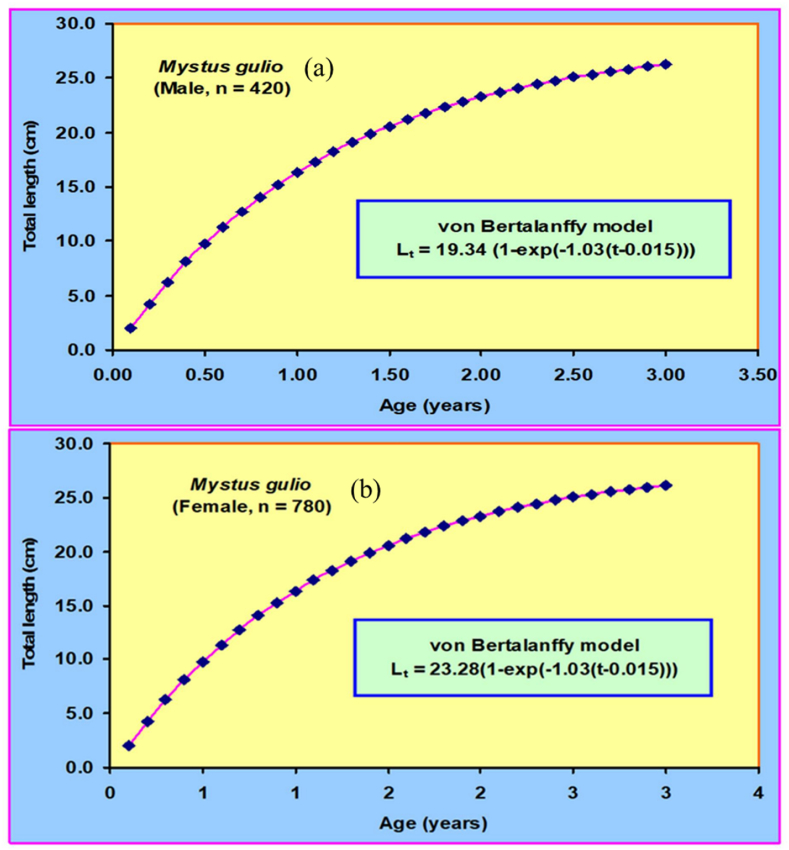
The von Bertalanffy growth curve based on length for the (a) male (*L*_*∞*_ = 19.34 cm, *K* = 0.94 year^−1^, *t*_*0*_ = 0.019 years) and (b) female (*L*_*∞*_ = 23.28 cm, *K* = 0.81 year^−1^, *t*_*0*_ = 0.025 years) of *Mystus gulio* in the coastal waters, southwestern Bangladesh as superimposed on the restructured total length-frequency histogram.

Growth parameters assessments were carried out by add in solver model tool since at least a consistent cohort during the sample period in both sex of population could not be traced in this experiment. The application of the Powell-Wetherall approach to analyze the combined length-frequency data of male *M. gulio* resulted in an initial TL estimate of 18.40 cm, accompanied by a *Z/K* ratio of 3.36 whereas the recorded maximum total TL measurement reached 18.30 cm, while the estimated maximum TL was 19.34 cm. The Powell-Wetherall technique, on the other hand, yielded a primary TL value of 18.30 cm and *Z/K* of 2.67 from the pooled LFD data of female *M. gulio* (Fig. 4(a and b)). The K-Scan analysis was performed to improve the estimate of growth characteristics for the male *M. gulio* population using the starting seed value of TL = 18.40 cm, an optimized and the following characteristics of a von Bertalanffy growth curve were successfully calculated: For male TL = 19.34 cm, *K* = 0.94 and 0.81 year ^−1^, *t*_*0*_ = 0.019 years, separately and the growth curve computed using these specific parameters is visually presented over the restructured TL distribution in Fig. 5. For the female *M. gulio* population using the starting seed value of TL = 18.30 cm, an optimized. A von Bertalanffy growth curve characterized by the parameters TL = 23.28 cm and *K* = 0.81 year^−1^, *t*_*0*_ = 0.025 years (Fig. 5(a and b)), was generated to describe the growth pattern of the studied population depicted the female growth curve apply to over the redesigned total length-frequency histogram. The recorded TL measured 22.10 cm, which fell slightly short of the anticipated maximum TL of 23.28 cm.

### 3.4 Size at first sexual maturity (*L*_*m*_)

The size at first sexual maturity *(L*_*m*_*)* was 10.89 cm for male and 12.95 cm for female species. (Table 3).

**Table 3:** Growth parameters (*L*_*∞*_ and *K*) and mortality parameters (*Z, M, F*) of *Mystus gulio* (Hamilton, 1822) based on length in the coastal waters southwestern, Bangladesh.

Description of Parameters	Values	
Growth and reproduction	Male	Female
Asymptotic length (*L*_*∞*_)	19.34 cm TL	23.28 cm TL
Growth coefficient (*K*)	0.94 year^−1^	0.81 year^−1^
Growth performance indexes (Ø')	2.55	2.64
Length at first recruitment	8.00 cm	9.50 cm
Age at zero length (*t*_*0*_)	0.019 years	0.025 years
Size at sexual maturity (*L*_*m*_)	10.89 cm	12.95 cm
Age at first sexual maturity (*t*_*m*_)	0.90 years	0.81 years
Longevity (*t*_*max*_)	2.97 years	2.90 years
**Mortality parameters**		
Total mortality (*Z*)	4.30 year^−1^	3.57 year^−1^
Natural mortality (*M*)	1.55 year^−1^	1.59 year^−1^
Fishing mortality (*F*)	2.75 year^−1^	1.98 year^−1^

### 3.5 Growth performance index and longevity

Based on asymptotic length, the calculated growth performance index for both specimens of *M. gulio* individuals was 2.55 and 2.64, respectively. Moreover, it was determined that the lifespan (*t*_*max*_) of males was approximately 2.97 years, while females exhibited a slightly lower lifespan of 2.90 years.

### 3.6 Length at recruitment

**Fig. 6:**
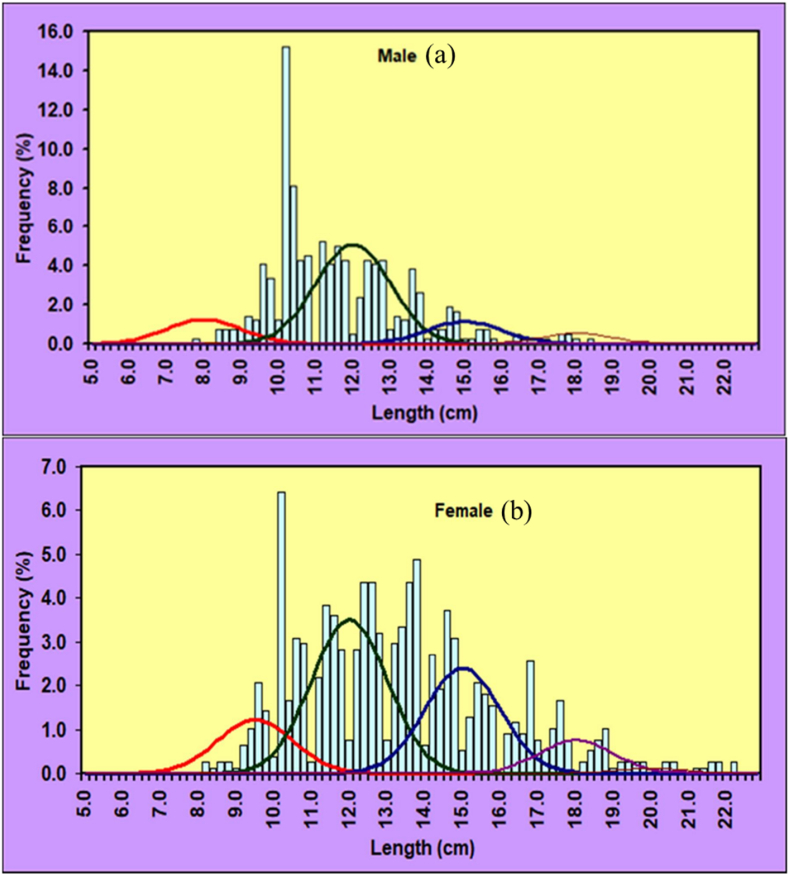
Length at first recruitment pattern for the (a) male and (b) female of *Mystus gulio* in the coastal waters, southwestern, Bangladesh through Excel-add-in- solver program.

In the Malancha River, the recruitment pattern of *M. gulio* remained consistent year-round, with a notable peak occurring during the 8.00, 9.50 cm size ranges for both individuals, as illustrated in Fig. 6(a and b).

### 3.7 Stock assessment

**Fig. 7:**
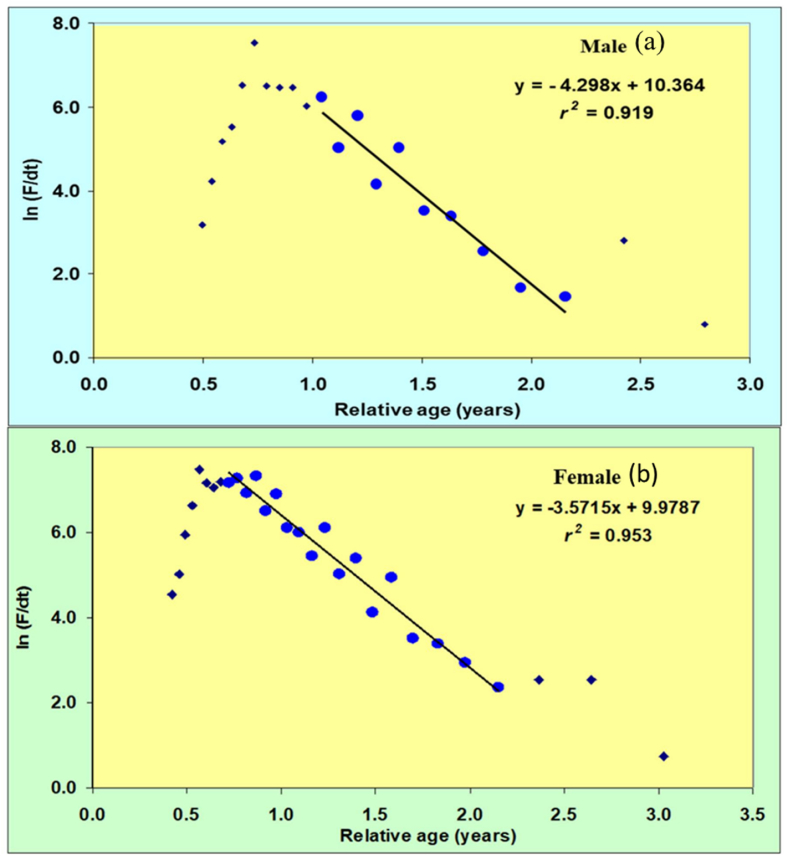
Length-converted catch curve for the (a) male and (b) female of *Mystus gulio* in the coastal waters, southwestern, Bangladesh. Data included in the regression are shown as blue solid points.

Fig. 7(a and b) visually illustrate the comprehensive mortality data obtained through the analysis of the length-converted catch curve slope, encompassing both sex of *M. gulio* specimens. The natural mortality of specimens was *M* = 1.55 year^−1^, 1.59 year^−1^ for males and females, indicating a slightly higher rate among the latter. Therefore, the male *F* was determined as 2.75 years^−1^, while the female *F* was calculated as 1.98 years^−1^. The analysis of the length-converted catch curve revealed an estimated as *Z* = 4.30 year^−1^ and 3.57 year^−1^ for male and females. (Table 3).

### 3.8 Maximum sustainable yield

**Fig. 8:**
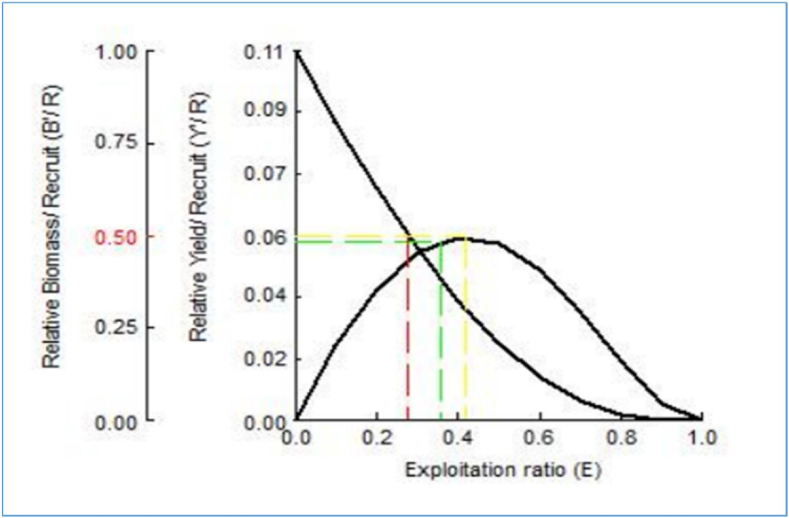
Yield-per-recruit and average biomass per recruit models, showing levels of yield index of *Mystus gulio* is in the coastal waters, southwestern, Bangladesh.

**Fig. 9:**
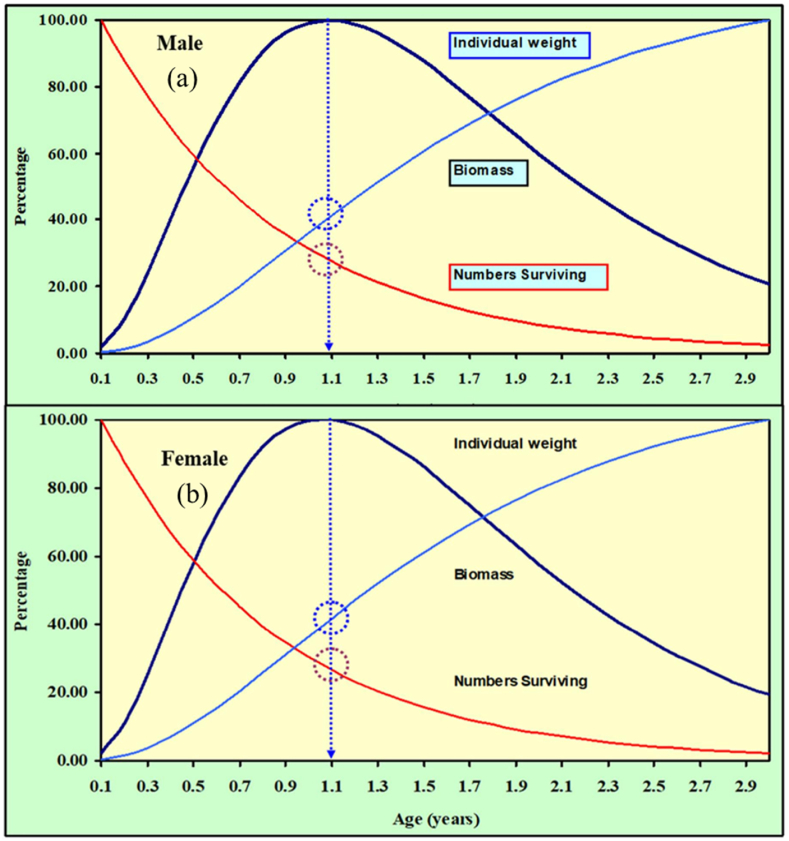
Percent of biomass, survival rate and individual weight for the (a) male and (b) female of *Mystus gulio* in the coastal waters, southwestern, Bangladesh.

**Fig. 10:**
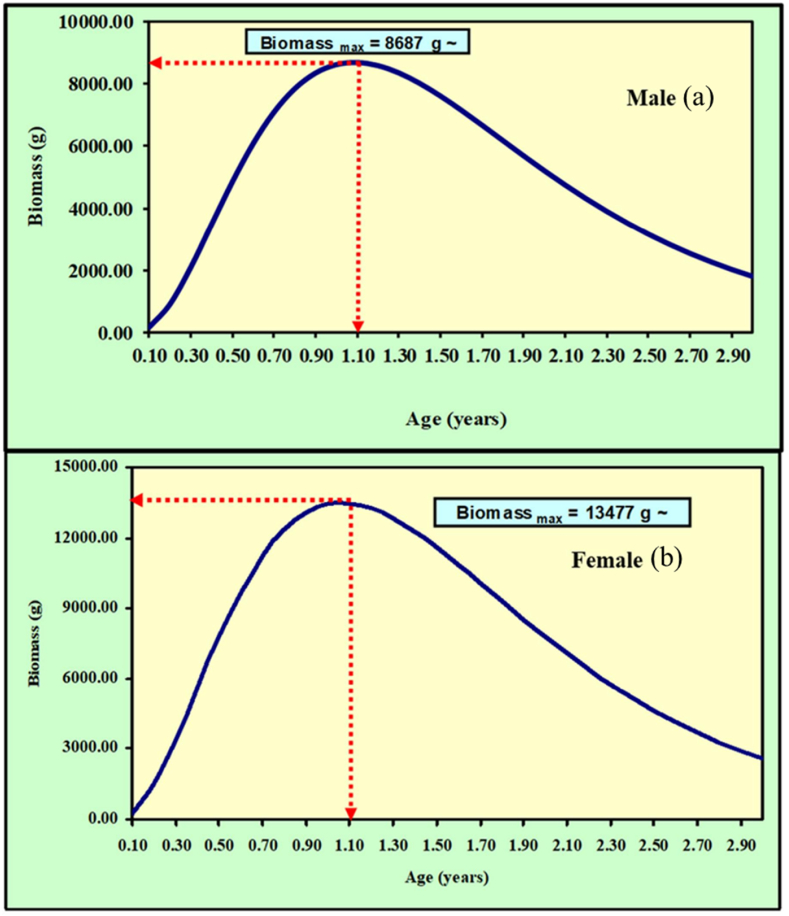
Biomass per recruit of the (a) male and (b) female of *Mystus gulio* in the coastal waters, southwestern, Bangladesh.

The maximum yield-per-recruit *(Y'/R)* of *M. gulio* in the Malancha River was 0.480, with male and female overexploitation being 0.16 and 0.14 respectively. From *(Y′/R)* analysis, the assessed values of *E* (Exploitation ratio), *E*_*max*_*, E*_*0.1*_*,* and *E*_*0.5*_ were 0.639, 0.421, 0.355 and 0.278, individually (Table 4; Fig. 8). Maximum biomass (relative) was found when male population reached at 1.1 year. On the other hand, maximum biomass was found when female population reached at same age 1.1 year (Fig. 9(a and b)). Maximum biomass (relative) was 8687g for male and maximum biomass was 13477g for female as illustrated in Fig. 10 (a, b). Utilizing the FiSAT II length-structured virtual population analysis approach, the total steady state biomass (SSB) was estimated 151.04 metric tons (Table: 4, Fig. 11). Subsequently, the *MSY* of *M. gulio* was expected at 67.968 metric tons.

**Table 4:** Fishery (*E, E*_*max*_*, Y′/R*, SSB and *MSY*) parameters of *Mystus gulio* (Hamilton, 1822) based on length in the coastal waters southwestern, Bangladesh.

Description of Fishery Parameters	Values
Exploitation ratio (*E)*	0.63
*E*_*max*_	0.42
*E*_*0.1*_	0.35
*E*_*0.5*_	0.27
Maximum yield-per-recruit (*Y′/R*)	0.48
Steady State Biomass (SSB)	151.04 MT
Maximum sustainable yield (*MSY*)	67.968 MT

**Fig. 11:**
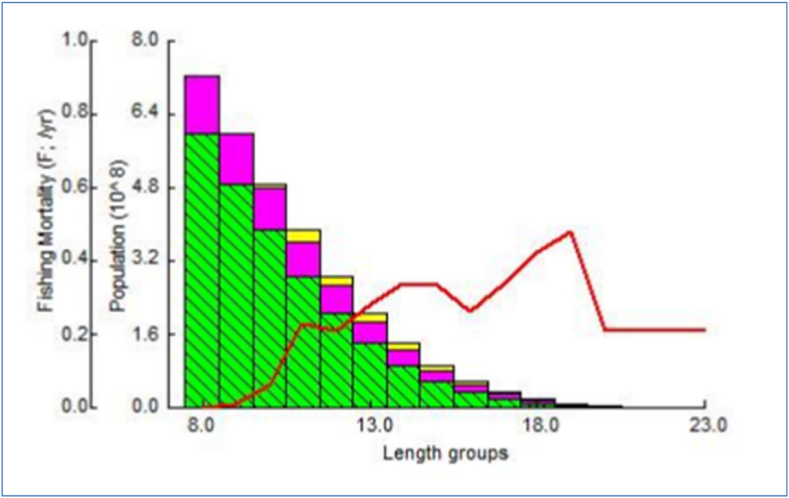
Length-structured virtual population analysis of *Mystus gulio* in the coastal waters, southwestern, Bangladesh.

## 4 Discussion

Stock assessments serve as a fundamental management approach essential for comprehending the growth, recruitment, mortality, maximum sustainable yield, and exploitation rate of fish [61,62]. Notably, research on the stock assessment of *M. gulio* is absent in Bangladesh and elsewhere. In our study, we meticulously sampled a substantial number of specimens (1200) over twelve successive months, utilizing local gears, including gill nets, in the coastal waters of Southwestern Bangladesh. Throughout the sampling period, capturing any *M. gulio* with a total length (TL) of less than 7.80 cm proved to be challenging, regardless of the population or the choice of fishing gear [63].

In current study, 1200 individuals with varied sizes were recorded to estimate the length frequency distribution. The maximum population was estimated in this research during the month of September and in the length class of 10.99–11.99 cm. There is very little information available on the length frequency distribution of *M. gulio* in coastal water therefore comparing the results of this study is not feasible.

The male *M. gulio* exhibited a TL range from 7.80 to 18.30 cm, whereas the female counterparts showcased a TL range extending from 9.30 to 22.10 cm. It is worth noting that individuals measuring below 7.80 cm TL and those surpassing 22.10 cm TL were not captured during the study period. Remarkably, the observed pinnacle of *M. gulio*'s length, recorded at 22.10 cm, surpassed the previously documented maximum lengths in other regions: Chilika Lagoon, India (TL = 21.50 cm) [25], and the Rupsha River, Bangladesh (TL = 17.20 cm; [64]. Accurate determination of the maximum lengths of fishes holds crucial significance in estimating both asymptotic length and growth coefficient, playing a pivotal role in the realm of fisheries resource planning and management strategies [65].

Generally, the overall *a* value for these specimens were found to be 0.049. However [25], reported different *a* value for this species as 0.0059 and 0.0106, for both sexes, respectively. Likewise, recorded *a* value of 0.0219 [24], for the combined sexes, a result that diverges from the literature. The *b* values within LWRs are considered optimal when falling between 2.5 and 3.5, as recommended by Froese [48]. Our analysis of the growth pattern reveals a combination of positive allometric growth (*b* > 3) and negative allometric growth (*b* < 3), as outlined by Tesch [66]. In the case of *M. gulio*, the allometric coefficient *b* value was determined to be 2.50 for male and 2.53 for female specimens. This result indicates negative allometric growth for both individuals. For instance Ref. [24], reported positive allometric growth (*b* = 3.11) in the Ganges River, Bangladesh [25], observed an isometric growth pattern (*b* = 3.03) in the Chilika Lagoon, India, and [67], noted negative allometric growth (*b* = 2.20) in the Narreri Lagoon, Pakistan. It is of utmost importance to acknowledge that growth patterns in fish populations can exhibit fluctuations owing to various factors, including habitat conditions, seasonal variations, stomach fullness levels, gonad maturation, health status, preservation techniques, and differences in the categorization of length classes within collected samples, as underscored [66,68]. Regrettably, these factors were not considered within the scope of this study. In contrast, the parameter *b* remains relatively stable and does not exhibit significant variations over the course of the year, as noted [69].

The growth pattern of *M. gulio* was most accurately described by the von Bertalanffy model, a widely employed framework in fishery biology renowned for effectively portraying fish growth patterns [70]. By utilizing *L*_*∞*_ = 18.40 cm as a seed value for male *M. gulio*, the estimated asymptotic length was found to be *L*_*∞*_ = 19.34 cm. Similarly, for females, using *L*_∞_ = 19.34 cm as a seed value, the estimated asymptotic length was calculated *L*_*∞*_ = 23.28 cm. Furthermore, the parameter *t*_*0*_ was computed as 0.019 years for male and 0.025 years for female specimens utilizing the King formula [71] and female had the higher value of this parameter than male. Male *M. gulio* reached an asymptotic body weight of 72.34 g with a growth coefficient of 0.83 per year, while female *M. gulio* attained 94.78g as asymptotic weight with a growth coefficient of 0.81 year^−1^. Due to absence of previous studies on this specific issue, a direct comparison with the present findings was not possible.

The growth performance index (*Ø*'), which serves as a measure of the relative well-being of aquatic organisms within their ecosystem, carries significant importance in evaluating their overall condition [72].

In this investigation, the computed growth performance index for male and female *M. gulio* was 2.59 and 2.75, respectively, based on asymptotic length. Consequently, the results indicate that females exhibited faster growth than males. The variations observed in the growth performance index between the male and female can be attributed to the previously mentioned life-history strategy, which elucidates the size distinctions inherent to male and female *M. gulio* individuals [73]. Furthermore, the longevity of both specimens was estimated 2.97 and 2.90 years, respectively, in the Malancha River. These variations in longevity are mainly influenced by environmental variables, with water temperature playing a pivotal role [74,75]. During this investigation, the recruitment pattern of *M. gulio* in the Malancha River was found throughout the year, with the peak observed at lengths of 8.00 cm and 9.50 cm for both individual, respectively. Unfortunately, there is no existing literature available on the recruitment of this species, which makes it challenging to compare the present findings with others.

Stock assessments provide crucial information to fisheries managers, aiding in the regulation and management of fish stocks by describing their past and current status. Natural mortality assumes a pivotal role among the critical variables in fisheries stock assessment and management. Its magnitude is directly related to stock productivity, achievable yields, optimal exploitation rates, management decisions, and reference points. In fisheries population dynamics, fishing mortality is another essential parameter that accounts for the loss of fish from a stock due to fishing activities. The age at maximum yield per recruit estimated at 1.06 years for both males and females. Using the length-converted catch curve analysis, total mortality *(Z)* was determined to be 4.30 year^−1^ and 3.57 year^−1^, *M* = 1.55 year^−1^ and 1.59 year^−1^ for both specimens of *M. gulio*. As a result, the fishing mortality *(F)* was documented as 2.75 year^−1^ for both males and females. From these estimations of instantaneous fishing and total mortalities, the exploitation rate (*E*) was derived and found 0.639 of *M. gulio* within the Malancha River. However, due to the absence of available literature, a direct comparison of these findings with other studies was not feasible.

The relative yield per recruit analysis reveals that the maximum yield per recruit of *M. gulio* was achieved *((Y′/R))* 0.48 (48 %), *E*_*max*_, *E*_*0.1*_, *E*_*0.5*_ and *MSY* were 0.42, 0.35, 0.27 and 67.968 MT. However, the current exploitation rates in the Malancha River of specimens, recorded at 0.63, indicating that *M. gulio* is being overexploited. Although classified as a species of least concern, this particular organism is not widely abundant within the Malancha River and continues to confront a range of threats that could potentially result in its population decline. Therefore, it is crucial to implement appropriate measures to conserve this species in its natural habitat. As there were no previous studies on these aspects, a direct comparison with other findings was not possible due to the lack of available literature. Moreover, the reasons for the declining biodiversity and the threats to *M. gulio* in the Malancho River encompass a range of factors. The population of fish species was slightly exploited since the exploitation rate was more than 50 %; hence, reduced fishing effort was required to maintain the population [76]. This over-exploitation due to excessive fishing activities, environmental degradation resulting from human impacts, the use of destructive fishing gear that harmfully affects the fish population, destruction of fry and fingerlings, the disruption of breeding grounds, the spread of diseases affecting the fish, siltation leading to unfavorable habitat conditions, and various ecological changes in its natural habitat.

## 5 Conclusion

The study focuses on elucidating the growth parameters, pattern, mortality, recruitment, exploitation rate, and biomass of *M. gulio* in the coastal waters of southwestern Bangladesh. The findings revealed over exploitation levels of 16 % for males and 14 % for females of *M. gulio*. If fishing activity continues unrestricted within this range, overfishing may emerge as the primary threat to the wild population of *M. gulio*. To ensure sustainable management, the study proposes a management approach that considers both biological and environmental aspects. These management recommendations are suggested to be implemented for *M. gulio* stocks in the coastal waters southwestern Bangladesh.

## Funding

The authors would like to thank National Science and Technology Fellowship for supporting the current study.

## Data availability statement

Available upon reasonable request.

## Ethics approval

All the procedures followed in this study were prescribed by the ethical approval committee of the Faculty of Agriculture, 10.13039/501100016173University of Rajshahi (FoA-RU: 003–2017). Informed consent is not applicable.

## Consent to participate

Not applicable.

## CRediT authorship contribution statement

**Obaidur Rahman:** Writing – original draft, Methodology, Formal analysis, Data curation, Conceptualization. **Taiba Akter Laboni:** Writing – original draft, Conceptualization. **Mst. Shahinur Khatun:** Writing – original draft, Conceptualization. **Md. Ashekur Rahman:** Writing – original draft, Formal analysis, Data curation, Conceptualization. **Md. Akhtarul Islam:** Writing – original draft, Formal analysis, Data curation, Conceptualization. **Md. Mizanur Rahman:** Writing – original draft, Data curation. **Most. Farida Parvin:** Writing – review & editing, Writing – original draft, Conceptualization. **Md. Joynal Abedin:** Writing – original draft, Data curation. **Md. Yeamin Hossain:** Writing – review & editing, Supervision, Project administration, Formal analysis, Conceptualization.

## Declaration of competing interest

The authors declare the following financial interests/personal relationships which may be considered as potential competing interests: Obaidur Rahman reports financial support was provided by Ministry of Science and Technology, Bangladesh. If there are other authors, they declare that they have no known competing financial interests or personal relationships that could have appeared to influence the work reported in this paper.
